# Proteins as Nano-Carriers for Bioactive Compounds. The Case of 7S and 11S Soy Globulins and Folic Acid Complexation

**DOI:** 10.3390/polym10020149

**Published:** 2018-02-05

**Authors:** María Emilia Ochnio, Jimena H. Martínez, Mariana C. Allievi, Marcos Palavecino, Karina D. Martínez, Oscar E. Pérez

**Affiliations:** 1 Departamento de Química Biológica, Facultad de Ciencias Exactas y Naturales, Intendente Güiraldes, s/n, Ciudad Universitaria, Buenos Aires CP 1428, Argentina; memilia.ochnio@gmail.com; 2 Consejo Nacional de Investigación Científica y Técnicas de la República Argentina IQUIBICEN-CONICET, Universidad de Buenos Aires, Departamento de Química Biológica, Facultad de Ciencias Exactas y Naturales, Intendente Güiraldes, s/n, Ciudad Universitaria, Buenos Aires CP 1428, Argentina; jhebemartinez@gmail.com (J.H.M.); marianaallievi@gmail.com (M.C.A.); palavecino.marcos@gmail.com (M.P.); 3 Consejo Nacional de Investigación Científica y Técnicas de la República Argentina, CONICET, Universidad de Buenos Aires, Departamento de Industrias, Facultad de Ciencias Exactas y Naturales, Intendente Güiraldes, s/n, Ciudad Universitaria, Buenos Aires CP 1428, Argentina

**Keywords:** 7S and 11S globulins, nano-complexation, folic acid, *Lactobacillus casei* BL23

## Abstract

Isolated 7S and 11S globulins obtained from defeated soy flour were complexated with folic acid (FA) in order to generate nano-carriers for this important vitamin in human nutrition. Fluorescence spectroscopy and dynamic light scattering were applied to follow the nano-complexes formation and for their characterization. Fluorescence experimental data were modeled by the *Stern-Volmer* and a *modified double logarithm* approach. The results obtained confirmed static quenching. The number of binding sites on the protein molecule was ~1. The values obtained for the binding constants suggest a high affinity between proteins and FA. Particle size distribution allowed to study the protein aggregation phenomenon induced by FA bound to the native proteins. Z-average manifested a clear trend to protein aggregation. 11S-FA nano-complexes resulted in more polydispersity. ζ-potential of FA nano-complexes did not show a remarkable change after FA complexation. The biological activity of nano-complexes loaded with FA was explored in terms of their capacity to enhance the biomass formation of *Lactobacillus casei* BL23. The results concerning to nano-complexes inclusion in culture media showed higher bacterial growth. Such a result was attributed to the entry of the acid by the specific receptors concomitantly by the peptide receptors. These findings have technological impact for the use of globulins-FA based nano-complexes in nutraceutical, pharmaceutical and food industries.

## 1. Introduction

Proteins are a versatile class of biopolymers whose function properties are dictated by their amino acid composition. In fact, proteins are polymers of amino acid [1]. Between these biopolymers, some proteins are widely used in pharmaceutical, nutraceutical and food formulations because they have high nutritional value and are generally recognized as safe. Numerous excellent articles highlight the functional properties of proteins including emulsification, gelation, foaming and water binding capacity [2,3] which are crucial for technological applications.

Soy globulins are storage proteins accounting for about 50–90% of seed proteins. They are grouped into two types according to their sedimentation coefficients—β-conglycinin (a 7S type globulin) and glycinin (an 11S globulin). The first is a trimeric globular glycoprotein with a molecular weight (MW) between 150–200 kDa. This globulin is composed of a combination of subunits with similar amino acid sequences, with three majorities: α (MW 57–72 kDa, isoelectric point (pI) of 4.9), α’ (MW 57–68 kDa, pI 5.2) and β (MW 42–52 kDa, pI 5.7–6.0) [4] and a γ minority subunit similar in size to the β subunit. Its complex quaternary structure is stabilized mainly by hydrophobic and hydrogen bond interactions [5].

On the other hand, 11S Glycinin is an oligomeric and compact globular protein, it has a MW ranged between 300–380 kDa and a pI 4.6 [6]. It is an hexamer in which, each of the not identical 6 subunits, is composed of an acid polypeptide “A” (MW 34.8–45 kDa, pI 4.75–5.40) and a basic polypeptide “B” (MW 19.6–22 kDa, pI 8.05–8.5) [7], both are synthesized by the same precursor that suffers a post-translational modification being united by a disulfide bond.

From a physicochemical point of view, a feature of soy proteins is the pH and ionic strength dependence of the molecular conformation and the associated functional properties, solubility between them [8,9]. From a nutritional point of view, soy proteins contains all the essential amino acids and in sufficient quantities to meet the protein intake requirements according to the needs of age and heath conditions of consumers [10]. This makes it a substitute for high quality, economical proteins, very important in the diet of countries where accessing proteins of animal origin for the human diet is expensive.

On the other hand, vitamins and antioxidants are rudimentary elements for human health as they assist the body to grow and develop. Furthermore, they are able to prevent diseases and to promote general health. Most of these bioactive agents are either produced in trifle amounts or not made in the body. Thus, vitamins need to be supplied from food products and through dietary supplements if needed [11,12]. Folates are a group of dietary compounds, which are chemically diverse and include all derivatives of tetrahydrofolic acid, a water-soluble vitamin of B complex. Folic acid (FA) and its bioequivalent folates are essential dietary components. This hydrophilic vitamin has been suggested to be effective in decreasing the risk for cardiovascular diseases [13] colon cancer [14], neurological illnesses such dementia and Alzheimer’s disease [15]. The vitamin is necessary for cell replication and has an important role in the prevention of neural tube defects (NTDs) [16]. It is most commonly known for its key role in women nutrition before conception, during pregnancy and lactation [17]. In aqueous solution at pH < 5, FA manifested particular features that make it unique, it is able to self-assemble into fine structures even at low concentrations such as 0.1% (*w*/*w*) through hydrogen bonds and stacking interactions [18].

Polymer-based delivery systems, proteins that trap molecules of interest within networks have been developed extensively for the biomedical and pharmaceutical sectors to protect and transport bioactive compounds to target functions [2,19]. Encapsulation is a promising and novel method for preserving the innate characteristics of vitamins over time [12]. This process includes coating or trapping a biomaterial or a combination into another element. In this context, soluble nano-complexes or nano-carriers of proteins with bioactive compounds as alpha-tocopherol, resveratrol, curcumin or even FA have been shown to delay at different extents, the degradation of these liable substances [20,21,22,23].

We hypothesize that 7S and 11S proteins are able to complexate with FA, constituting true nano-carriers for this crucial vitamin. According to the scientific literature, this is the first time that the construction of FA nano-carriers based on soy globulins via nano-complexation is reported. Therefore, the objective of the present contribution can be divided in three parts: (1) to generate nano-carriers based on 7S and 11S soy globulin for FA; (2) to characterize the nano-complexes in terms of size and ζ-potential and; (3) to explore the biological activity of nano-complexes in a microbiological model by their capacity to increase bacteria biomass formation. The approach has technological importance for the use of protein biopolymers in pharmaceutical, food and nutraceutical, industries, being the last one a booming area in recent years.

## 2. Materials and Methods

### 2.1. Materials

7S and 11S globulins were obtained from soybean defatted flour (Sanbra, S.A., Sao Paulo, Brazil), the protein content of which was 50%. Each one of globulin fractions were obtained by the Nagano method [24] with slight modifications introduced by [25] which consists of double time of centrifugation at each step. The final precipitated fractions were lyophilized and kept at −20 °C up to their use.

The final protein content in the 7S and 11S globulins isolated fraction, determined by the Kjeldahl method, was 96.1% and 97.9% (N × 6.25), respectively.

FA powder with a purity of >97%, was kindly given by Bago, Argentina. Both proteins and FA were used without further purification. Ultrapure water was always used.

Powder of each one of the protein fractions and FA was dissolved with analytical-grade Trizma [(CH_2_OH)_3_CNH_2_/(CH_2_OH)_3_CNH_3_Cl] at pH 7.0, supplied by Sigma (St. Louis, MO, USA) (99.5% and Milli-Q ultrapure water). All chemicals used for SDS-PAGE analysis were purchased from Bio-Rad Ltd. (Hong Kong, China).

### 2.2. 7S and 11S Mixed Solution Preparation

A stock solution containing 1%, *w*/*w*, of FA was prepared and diluted in the same buffer. Lyophilized 7S and 11S globulins fractions and FA were dissolved separately at room temperature under agitation. The solutions were freshly prepared, filtered through 0.45 and 0.22 μm microfilters (Whatman International Ltd., Maidstone, UK) and kept for 24 h at 4 °C to achieve the complete hydration of the molecules [23]. The required final concentrations of 7S and 11S soy globulins and FA to generate both types of nano-complexes were produced by mixing the appropriate volume of the double concentrated solutions of each protein and vitamin at pH 7 Globulins and FA concentrations in the solutions boson variated in according to the conditions demanded by the applied technique.

### 2.3. SDS-PAGE Electrophoresis

Acrylamide running gels of 12% with 10% *w*/*w* of SDS were run for 60 min at constant 150 V in a Mini-Protean Tetra Cell unit. A continuous buffer system was used, consisting of premixed electrophoresis buffer. The weight of deposed protein was 40 μg. The buffer contained 25 mM Tris and 192 mM glycine. Afterwards, gels were stained by Coomassie brilliant blue R250 and individual gels were imaged with a Microtek 9800XL Plus scanner (Microtek, Carson, CA, USA) [26].

The quantification of 7S and 11S globulins and their contributing subunits were made by densitometry using Image J software (Bethesda, MD, USA). Thus, protein quantification resulted in 76.18% and 72.57% for 7S and 11S respectively over the total protein obtained.

### 2.4. Globulin-FA Interaction Studies: Fluorescence Measurements

The protein intrinsic fluorescence, due to tryptophan (Trp) and tyrosine (Tyr) residues, is sensitive to the fluorophore environment and thus potentially an indicator of changes in protein conformation. When excited at a wavelength (λ_ex_) of 295 nm, only Trp emits fluorescence while both Trp and Tyr emit when excited at 280 nm [20]. The intrinsic fluorescence of 7S and 11S globulins in solution was measured at room temperature at specified time intervals using a Cary Eclipse fluorescence spectrophotometer (Varian Inc., Hong Kong, China). Fluorescence emission spectra were recorded from 290 to 500 nm at λ_ex_ = 280 nm. Globulin concentrations, 7S or 11S, was 5.0 mM and FA concentration ranged from 0 to 20 mM in mixed solutions.

### 2.5. UV–Vis Absorbance Spectra

Absorbance spectra of 7S, 11S globulins solutions and for the globulins-FA mixed solutions were registered by a JASCO V650 (JASCO Inc., Tokyo, Japan). Spectra were obtained from 200 to 450 nm path-length quartz cuvettes. This assay gave complementary information to the fluorescence measurements (Section 2.4) and was performed to corroborate the fluorescence intensity quenching provoked by FA in mixed solutions [27]. As for fluorescence determinations, 7S or 11S solution concentration was 5.0 mM and FA concentration ranged from 0–20 mM in mixed solutions.

### 2.6. Globulins Aggregation: Studies of Particle Size and Zeta Potential

Particle size distribution and its derived parameters, main peaks, Z-average (Z_ave_) and ζ-potential measurements were carried out using a Zetasizer Nano-Zs particle analyzer (Malvern Instruments, Worcestershire, UK) under the conditions proposed by Pérez et al. [23]. All samples were analyzed with an angle of 173°, at 25 °C. Each measure consisted in 10 runs. The intensity distribution is determined using a multi exponential function (CONTIN) to fit the correlation data. In this type of analysis, the presence of more than one family of particle sizes is taken into consideration. These multi-exponential algorithms, for which there is no standard and which generally vary from manufacturer to manufacturer, will generate an intensity distribution and, using the Mie theory, a distribution normalized for the volume of the scattering particles [28]. Volume size distribution was considered to determine the relative importance of each peak. The following constants were used for measurements: RI: 1.333 for Trizma buffer; which was the dispersant, dielectric constant: 78.5, viscosity: 0.8872 cp, equilibration time was always 180 s. Samples were diluted with filtered double distilled water, equilibrated for 1 min inside the instrument before measurements. The Z_ave_, particle diameter was also determined through DLS measurements, whose data was translated into Z_ave_ sizes using the Stokes Einstein equation [29]. The breadth of the peak distribution is typically parameterized by the polydispersity index (PDI) in particle size distribution studies. This parameter has the appropriately pleasing property that it is unity for a perfectly monodisperse system and increases as the volume distribution increases in breadth (M.I. Ltd., Worcestershire, UK, Protein Characterization Using Dynamic & Static Light Scattering Techniques from Malvern Instruments).

ζ-potential of particles was collected over at least 12 sequential readings to determine the electrophoretic mobility of the samples. The ζ-potential of the particles was calculated using the Huckel model based on the rationale that, since the investigated nano-complexes are close to the dimensions of the globular proteins, the Huckel is more suitable than the Smoluchowski model.

For particle size and ζ-potential analysis, 7S concentration was 5.5 mM and 11S protein concentration was equal to 3.0 mM (equivalent to 0.1%, *w*/*w*, for both globulins). Two FA concentrations were assessed in mixed solutions, 4.5 and 7.0 mM (equivalent to 0.2% and 0.3%, *w*/*w*, respectively).

### 2.7. Determination of FA in Samples

The amount of FA bound to 7S or 11S globulin fractions was determined by the difference between the amounts of FA initially added to the mixed solutions minus the FA not bound or free FA. This was the amount of FA in the supernatant after ultracentrifugation and concentration with 10k (Centricon AmiconR Ultra-4, Millipore, Ireland). 2 mL of each mixed samples were centrifuged into the filters, which weights had been previously taken. Filters were put into 4 mL plastic tubes. Centrifugation was performed at 4500× *g* for 30 min at 24 °C. The permeated was collected and the filters weight was taken again. The remnant FA trapped into the filter matrix would be detectable by weight difference before and after centrifugation (<0.001 g).

Free FA contained into the permeated was determined by a microbiological assay according to the procedure explained below (Section 2.8.1) which corresponds to the standard methods of [30].

### 2.8. Microbiological Assays with Lactobacillus casei BL23

#### 2.8.1. Calibration Curve Construction for *L. casei* BL23

A modified method described by [31] was used. The strain, auxotrophic for FA, was grown at 37 °C for 24 h in de Man, Rogosa and Sharpe (MRS) medium. MRS medium (Biokar, Beauvais, France) pH = 6.5 contains 10 g tryptone L^−1^, 4 g yeast extract L^−1^, 8 g meat extract L^−1^, 5 g Na acetate L^−1^, 0.2 g MgSO_4_·7H_2_O L^−1^, 0.05 g MnSO_4_·4H_2_O L^−1^ and 1 mL Tween 80 L^−1^ and 20 g glucose L^−1^. The culture was then centrifuged (8000× *g* for 5 min, at 21 °C), washed with sterile distilled water twice, resuspended to its original volume with sterile physiological solution (NaCl 0.85%, *w*/*v*). The cell suspension was inoculated at OD_600_ = 0.130 in vitamin-free FACM medium (Folic Acid Casei Medium, Difco, Argentina) and incubated at 37 °C for 24 h. The culture obtained was washed and inoculated in FACM medium and different concentrations of commercial FA were added. The inoculated media with FA were incubated at 37 °C for 48 h and the optical density was read at 600 nm (OD)_600_.

A calibration curve for microbial growth as a function of FA concentration was generated. The media growth absorbance was the blank subtracted. Each point represents the mean of three replicates. The standard error was always <10%.

#### 2.8.2. FA Apparent Concentration [FA_app_] in Nano-Complexes

The procedure followed was the explained in Section 2.8.1 up to the last wash. After that, the culture was inoculated in FACM containing concentrations of 7S-FA or 11S-FA nano-complexes loading the equivalent amount of single FA. The FA content loaded by the nano-complexes ranged FA concentrations considered for the calibration curve range. As controls, pure globulins 7S or 11S simultaneously with the single FA were run and the growth of *L. casei* BL23 analyzed. FA was added at the concentrations used for the calibration curve construction.

Loading capacity for 7S-FA and 11S-FA nano complexes was determined by the Madziva et al. method [32].

LCFA (%) = [(FA_N_ − FA_S_)/FA_N_] × 100

where FA_N_ is the nominal FA concentration in the system and FA_S_ is the FA concentration after ultracentrifugation and determined by a microbiological assay (Section 2.8.1). The loading capacity was expressed in percentage.

### 2.9. Statistical Analysis

All the experiments were performed at least in triplicate. The model’s goodness-of-fit was evaluated by the coefficient of determination (*R*^2^) and the analysis of variance (ANOVA), using Statgraphics Plus 3.0. software (Statgraphics Technologies, Inc., The Plains, VA, USA).

## 3. Results

### 3.1. 7S and 11S Globulin Isolation

**Figure 1:**
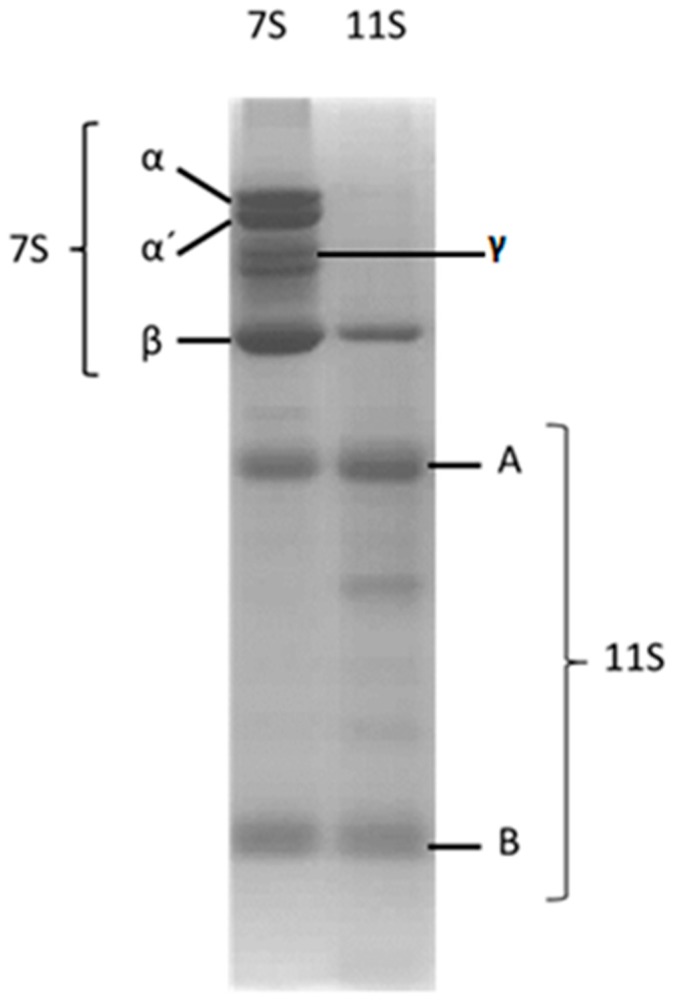
SDS-PAGE profile corresponding to 7S and 11S soy globulins isolated from defeated flour. The different subunits are pointed out. Proteins were dissolved in Trizma buffer, pH 7, *I* = 0.05 M.

The principal storage proteins for soybean were isolated from the defeated flour. Figure 1 displays the SDS-PAGE profiles of the isolated fractions corresponding to both globulins. The mentioned subunits were detected by this technique and are pointed out in the figure. This protein pattern is coincident with previous reports [25]. As indicated in Figure 1, 7S globulin is an heterodimer constituted by three subunits: α (67 kDa), α’ (71 kDa) and β (50 kDa); γ conglicinin (140 kDa) which is present in a lower proportion [33]. On the other hand, 11S globulin was constituted by three acidic subunits A (~35 kDa) and three basic B (~20 kDa), determining a hexamer structure stabilized by S–S bounds [33].

### 3.2. Globulins and FA Interactions

The maximal FA concentration used was established in 20 μM as the acid suffered self-aggregation or auto-stacking with the consequent fluorescence auto-quenching at highest concentrations; which is coincident with previous reports [20].

**Figure 2:**
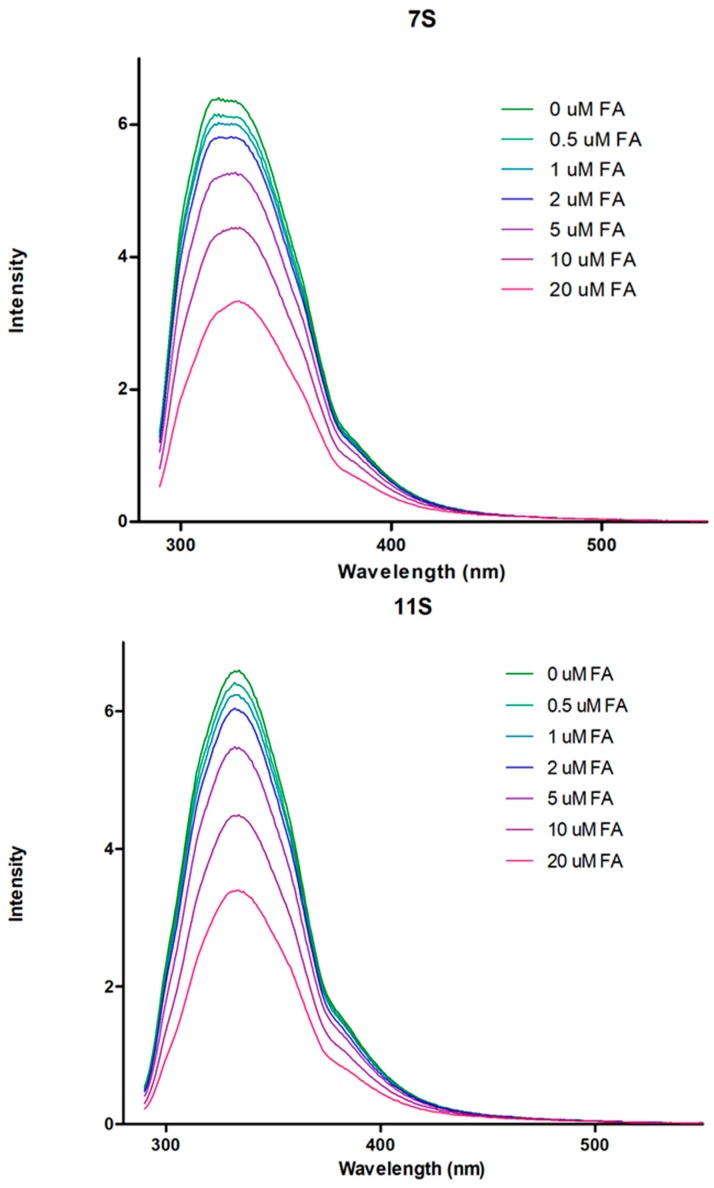
Fluorescence emission spectra for 7S and 11S globulin fractions, 5 μM for each protein and 0, 0.5, 1, 2, 5, 10 and 20 μM of FA. In Trizma buffer, pH 7.0 and *I* = 0.05 M. Temperature 25 °C.

The fluorescence of protein is predominantly attributed to the Try residue [34]. At this respect, Figure 2 shows the fluorescence spectra corresponding to 7S and 11S globulins at λ_ex_ = 280 nm superimposed with those corresponding to the mixtures 7S-FA and 11S-FA. Both globulins fractions presented a peak in the emission spectra at 330 nm. The wavelength for such peaks was not modified, indicating that the microenvironment of Try did not change for any globulin fraction in the presence of FA. Besides, from Figure 2 it can also be seen that the fluorescence intensity decreased as FA concentration increased. For instance, when FA concentration was equal to 20 μM in mixed aqueous solutions, the intrinsic proteins fluorescence intensity decreased 50% and 48% for 7S and 11S globulins, respectively. These results were a manifestation of the protein quenching induced by FA. The quenching phenomenon refers to processes that decrease the fluorescence intensity in a sample. Two kinds of quenching can be distinguished, the dynamic and the static type. The first one results from collisions between the fluorophore and the *quencher*, i.e., the compound that provokes the fluorescence intensity decrease. The quencher must diffuse and contact transiently the fluorophore during the half-life of the excited state. After contact, the fluorophore returns to its energy fundamental state without photon emission. This type of quenching occurs with no permanent changes in the molecules, there are not photochemical reactions. On the contrary, during static quenching a permanent complex is formed between the fluorophore and the quencher, which is not fluorescent [27].

**Figure 3:**
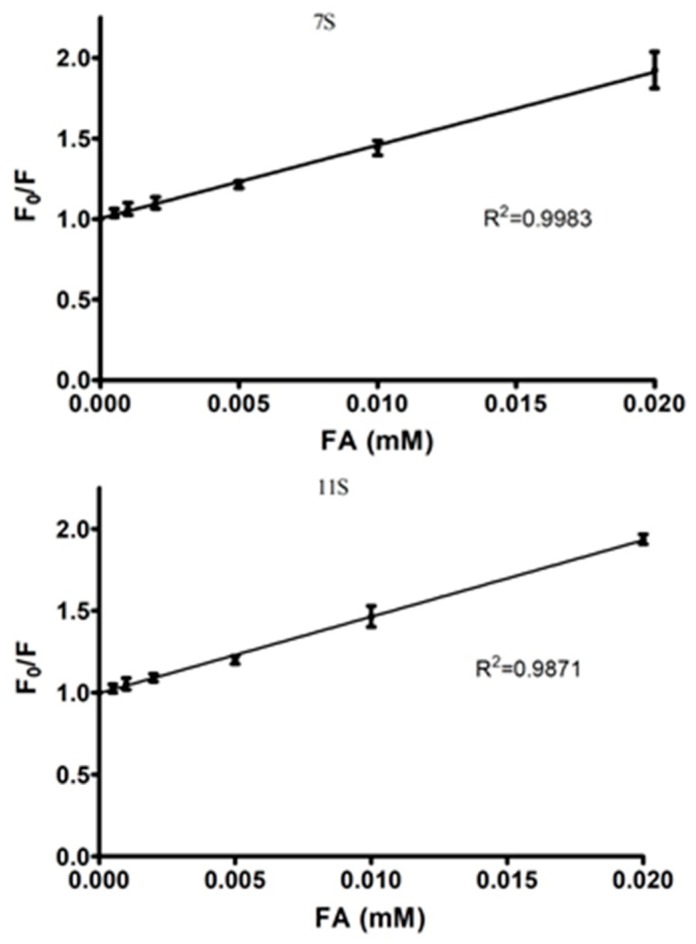
*F*_0_/*F* versus FA concentration (Stern Volmer model) for 7S and 11S globulins fractions. In Trizma buffer pH 7, *I* = 0.05 M. Temperature 25 °C.

The fluorescence experimental data can be analyzed by mathematical models. At this respect, the application of Stern-Volmer (Equation (1)) allows determining the dynamic quenching.

(1) $$\frac{F_{0}}{F}=1+k_{q}\tau_{0}[\mathrm{FA}]=1+K_{D}[\mathrm{FA}]$$

where, *F* and *F*_0_ are the fluorescence emission intensity with and without FA, respectively; *k_q_* is the quenching constant; *τ*_0_ is the half-life of the fluorophore fluorescence without FA (the quencher); [FA] is the FA concentration and *K_D_* is a constant equal to the reciprocal of FA concentration when the fluorescence intensity decrease 50% [20,27]. Figure 3 displays the lineal dependency of *F*_0_/*F* with FA concentration. The value of *K_D_* could be obtained from the slope of this relation. Under the conditions used in this work, *τ*_0_ acquires a value equal to 2.9 ns for the Trp residues. Therefore, *k_q_* was 1.57 × 10^13^ and 1.61 × 10^13^ M^−1^·s^−1^ for 7S-FA and 11S-FA mixed solutions, respectively. These values were higher than the constant admitted to dynamic quenching (1.27 × 10^10^ M^−1^·s^−1^) [35]. Therefore, the results would indicate that the fluorescence intensity changes detected for 7S and 11S globulins fractions could obey to protein-FA complexes formation as evidenced by static quenching. The *R*^2^ values obtained from the regression curves indicated that the model application was satisfactory. To analyze the fluorescence intensity dependence with the FA concentration, the Equation (2) can be used:

(2) $$\log[\frac{F_{0}-F}{F}]=\log K_{S}+n\log[\mathrm{FA}]$$

**Figure 4:**
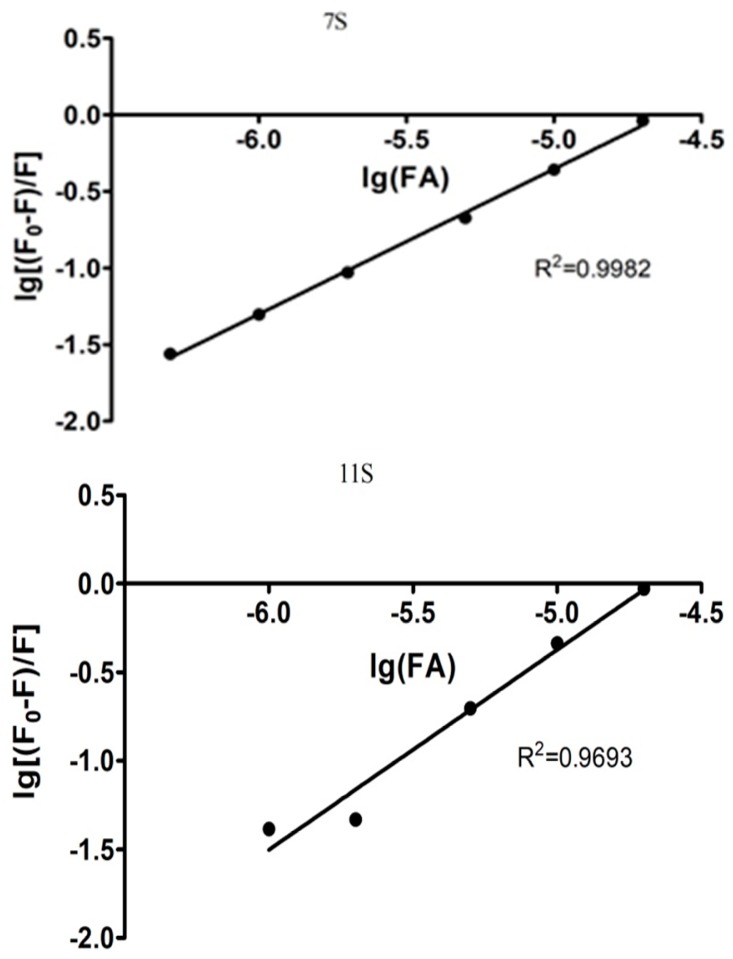
log[(*F*_0_ − *F*)/*F*] versus log[FA] for 7S and 11S globulins fractions. In Trizma buffer pH 7, *I* = 0.05 M. Temperature 25 °C.

*K_S_* is the association constant and *n* the binding sites for the ligand on the macromolecule [34,36]. Figure 4 shows the linear dependency of log[(*F*_0_ − *F*)/*F*] vs. log[FA]. The parameter *n* obtained from the line slope, was 0.95 (±0.02) and 1.13 (±0.12) for 7S-FA and 11S-FA, respectively. On the other hand, *K_S_* could be obtained from the intercept, resulting equal to 2.40 × 10^4^ and 1.87 × 10^5^ M^−1^, for 7S-FA and 11S-FA, respectively. The association constant was an order higher for 11S-FA complexes; which demonstrated that 11S globulin has a higher affinity for FA.

**Figure 5:**
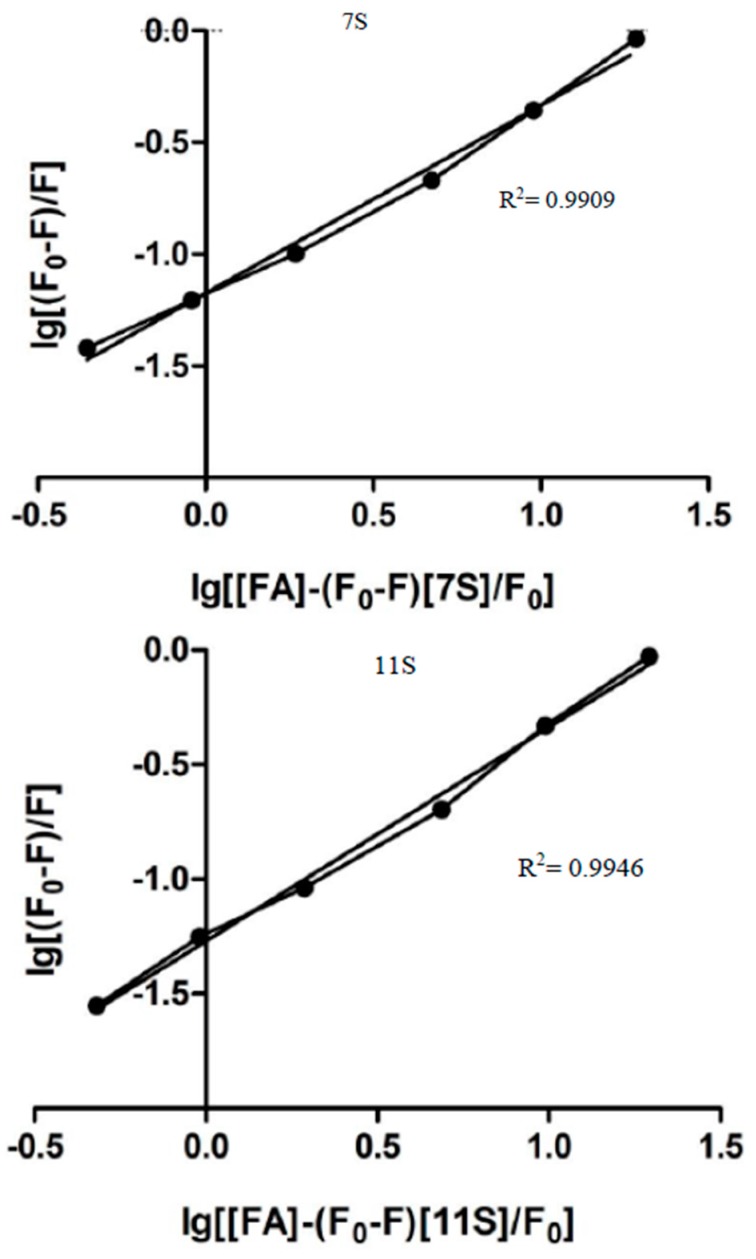
The modified Double logarithm regression curve for 7S and 11S globulins fractions. In Trizma buffer pH 7, *I* = 0.05 M. Temperature 25 °C.

The modified Double logarithm regression curve (Figure 5) was another approach applied here to find *K_a_* (analogous to *K_s_* in Stern-Volmer model) and the binding site number *n* [37,38,39]. Thus, Equation (3) describes the model.

(3) $$\frac{\log(F-F_{0})}{F}=n\log K_{a}+n\log[\frac{\left[Q]-(F-F_{0})[P\right]}{F_{0}}]$$

where *n* is the number of binding sites on the protein molecules and [*Q*] and [*P*] are the total concentration of quencher and protein, respectively. *n* can be obtained from the slope of linear regressions of the curve [log(*F*_0_ − *F*)/*F*] as a function of [log[*Q*] − (*F*_0_ − *F*)[*P*]/*F*_0_] and *K_a_*, the binding constant, was obtained from the intercept of the linear regression. *K_a_* and *n* values obtained by applying Equation (3) are summarized in Table 1. *K_a_* resulted practically identical for both proteins. The values corresponding to *n* resulted very similar when obtained by Stern-Volmer as by Double logarithm models. The high values of *R*^2^ demonstrated the goodness of fit for the modified Double log model application.

*n* values kept the trend observed with Stern-Volmer model, which resulted higher for 11S-AF (Table 1). *K_a_* adopted values practically identical for both proteins when the analysis was made by Double log approach. Although this parameter resulted one order of magnitude lower for 11S-AF system in comparison with that obtained by the Stern-Volmer model. In this respect, Wei et al. [40] exposed a detailed study about several macromolecules-ligand pairs. The authors observed differences for *K_a_* and *n* parameters when different models were applied and give possible explanations for such differences, which will be discussed later in the Discussion section.

### 3.3. Absorption Spectrum of Fluorophore

**Figure 6:**
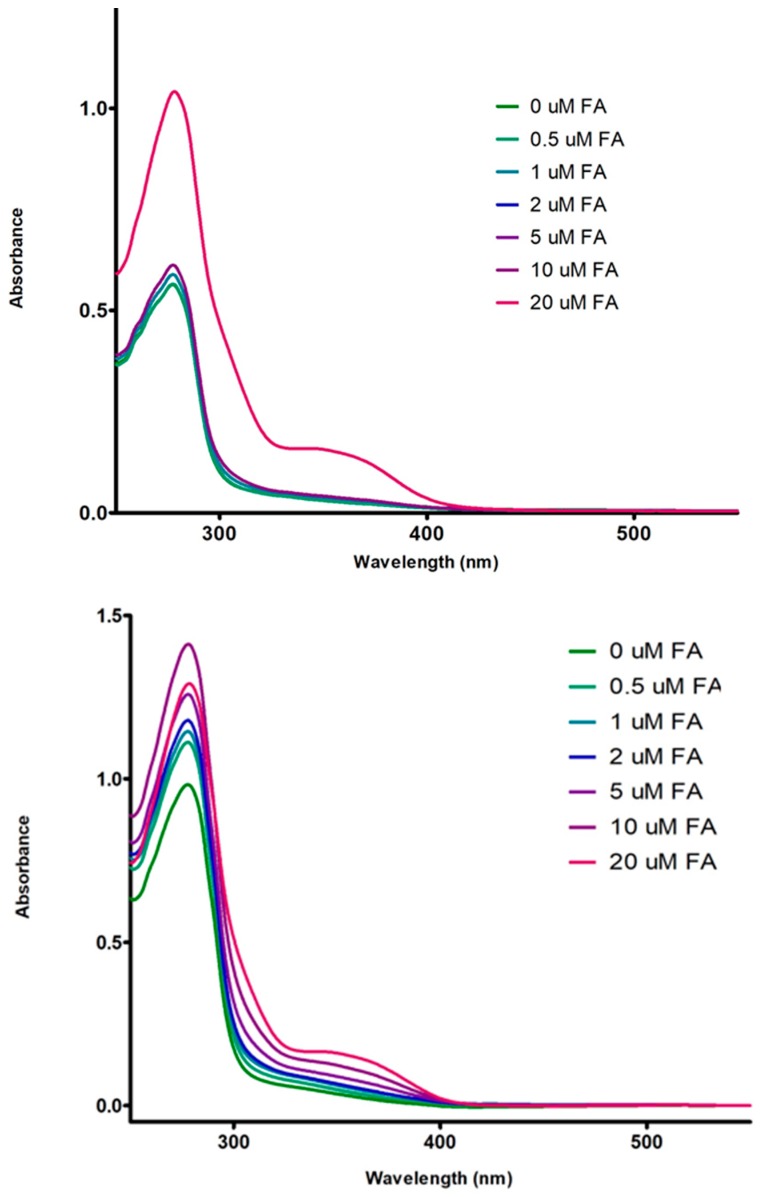
Absorption spectra for 7S (**top**) and 11S (**bottom**) globulin fraction (5 µM) in the presence of 0, 0.5, 1, 2, 5, 10 and 20 µM de FA. In Trizma buffer, pH 7, *I* = 0.05 M. Temperature 25 °C.

A useful complementary method to discriminate between static and dynamic quenching is the examination of the fluorophore absorption spectra in the UV region. Static quenching affects the fluorophores excited states of. When a complex formation occurs, a perturbation in the fluorophore absorption spectrum can be detected [27]. Figure 6 shows the adsorption spectra in the region UV-Vis for 7S-FA and 11S-FA mixed solutions containing different FA concentrations, which ranged 0–20 μM. The adsorption peaks were detected between 200 and 310 nm which obey to the Trp, Tyr and Phe presence. The peak observed at 250 nm was consistent with strong absorbance by fluorescent amino acids that absorb in this UV spectrum region. A maximum peak was observed at around 150 nm and a drop in absorbance at higher wavelengths, followed by an exponential decrease beyond 280 nm. No shift to higher wavelength was registered for the major peak in both cases, whose bands remain unaffected.

Samples presented a shoulder at the maximum FA concentration for 7S mixed systems corresponding to the influence of the vitamin. The mentioned shoulder was remarkable at lower FA concentrations for 11S-FA mixed solutions in comparison with 7S-FA system.

These results confirm the existence of static quenching as the absorbance of single protein decreased with the FA concentration increasing.

### 3.4. Analysis of Protein Aggregation: Particle Size and ζ Potential Determination

**Figure 7:**
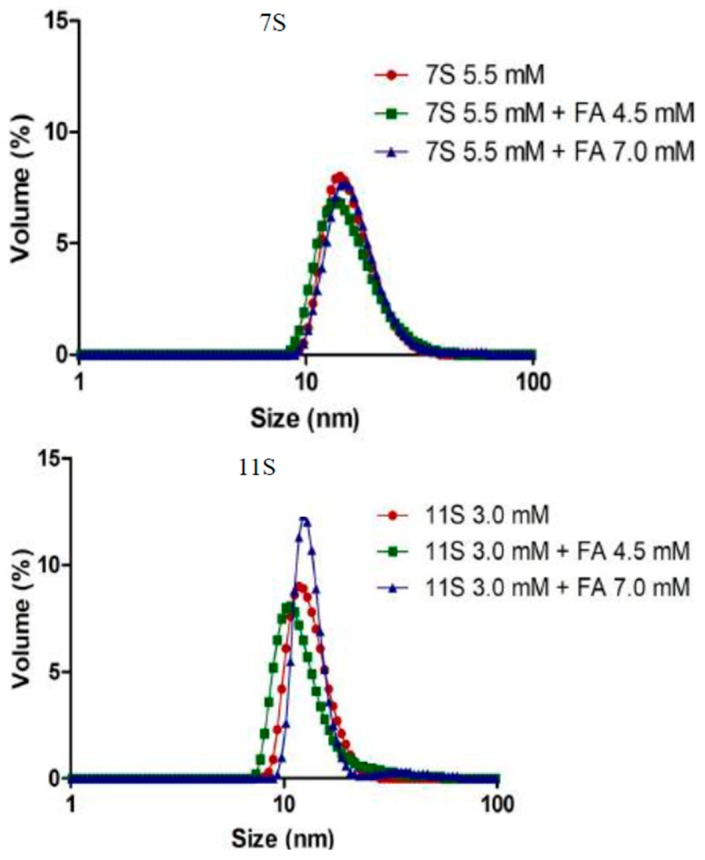
Particle size distribution for 7S (5.5 mM) and 7S-FA mixed systems with [FA]: 4.5 and 7.0 mM. 11S (3.0 mM) and 11S-FA mixed systems with [FA]: 4.5 and 7.0 mM. In Trizma buffer, pH 7, *I* = 0.05 M. Temperature 25 °C.

It can be seen in Figure 7 the particle size distribution obtained by DLS for 7S and 11S globulins and for globulin-FA nano-complexes. The Figure shows that the size distribution pattern was monomodal, which corresponds to the average of ten repetitions of the particle analysis present in each solution, which is expressed in volume (%). Single 7S globulin manifested a peak that ranged 10–35 nm with a maximum at 15 nm. On the other hand, single 11S globulin and 11S-FA mixed solutions manifested a narrower peak ranging from 10 up to 20 nm with its maximum at 12 nm. In general terms, a displacement to higher sizes resulted more remarkable from 11S-FA mixed systems. This result could be caused by the FA bounding to the globulin and the further aggregation of protein-FA loaded molecules. This result was not evident for the simple observation from particle size distribution, especially for 7S-FA nano-complexes. For this reason, the Z_ave_ parameter was analyzed. This parameter results useful to elucidate the possibility of protein aggregation and is commonly used to express particle size by DLS when the distribution is monomodal. Table 2 shows the average diameter values, Z_ave_, a parameter.

Z_ave_ comes from the cumulative analysis, specifically corresponding to the first part of the correlation function after fitted into a single exponential decay. Therefore, Z_ave_ size always provides with single value for every sample. Polydispersity index (PDI) is also shown in Table 2. The PDI for DLS typically depicts the intensity of light scattered by various fractions of the particles differing in their sizes and is calculated by (width/mean)^2^ for each peak [41].

The average diameter value arises when the DLS data is analyzed using the “cumulantes” method [42]. Since the calculations of this parameter are mathematically stable, the resulting Z_av_ is insensitive to noise and that makes it the preferred size parameter in DLS. The magnitude of Z_av_ increases as the particle size increases, therefore, it is a reliable parameter that globally characterizes a distribution.

The effect of the FA addition provoked gradual changes according to the Z_av_ values obtained for 7S fraction. However, a more pronounced averaged particle size increase was observed when the mixture was composed by the 11S fraction as indicated by a Z_av_ value of 51 nm, approximately. The vitamin union induced changes on the globulins molecules that subtly favor the hydrophobic interactions between 7S and 11S molecules loaded with FA.

According to the DLVO theory, high electrokinetic potentials result in high electrostatic repulsion between particles, which leads to high colloidal stability. One way to estimate the electrostatic forces involved in colloidal stability is by determining the electrokinetic potential, also known as potential ζ, which is the electric potential operating in the plane of a charged particle during electrophoretic movement [43]. The average of ten repetitions of the ζ potential for each globulin fraction, 7S and 11S and their combination with FA is reported in Table 2.

ζ potential values did not change remarkably with the increase in FA concentration. Both molecules have the same negative ζ-potential at the working pH. In this sense, the existence of non-covalent interactions is more likely, even though the electrostatic type interaction between local and specific groups could not be completely discarded.

The PDI, defined as the ratio of the square of the standard deviation to the square of the mean size [44] did not manifest statistically significant changes in the case of 7S-FA mixed system. However, when 11S-FA interactions were studied, an evident increase in PDI was found at 7.0 mM of the acid. The meaning of this finding could be understood as the existence of a variety of 11S aggregates induced by the FA bound, which in turn would be produced at the highest FA concentration and were visualized as a broader particle size distribution.

### 3.5. Analysis of Protein Aggregation: Molecular Weight Estimation

**Figure 8:**
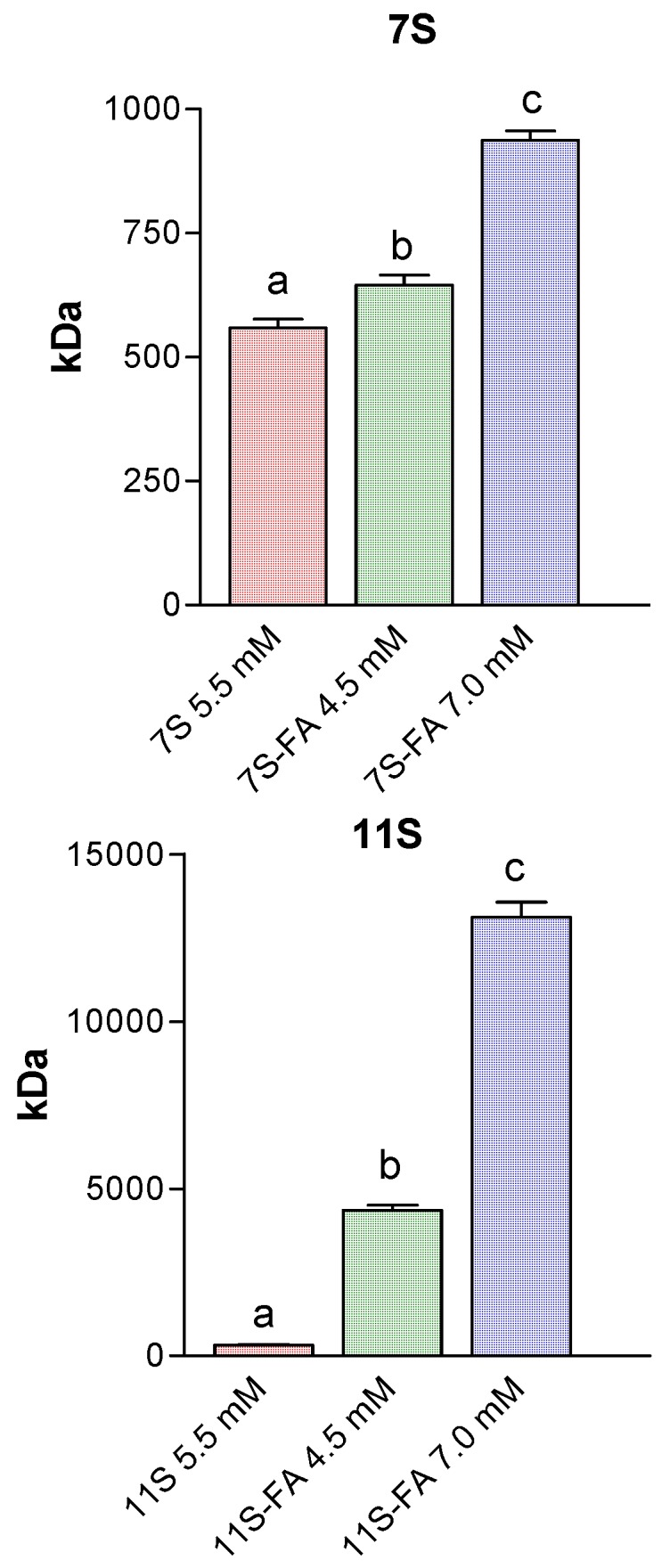
Molecular weight estimation for 7S (5.5 mM) and 7S-FA mixed systems [FA]: 4.5 and 7.0 mM; 11S (3.0 mM) and 11S-FA mixed systems [FA]: 4.5 and 7.0 mM. In Trizma buffer pH 7, *I* = 0.05 M. Temperature 25 °C.

Figure 8 shows the variation of 7S and 11S self-association state induced by FA complexation. It can be seen that the aggregates diameters resulted of higher sizes with FA concentration. The self-association state—i.e., aggregation state—of a protein can be influenced by intrinsic solution properties, such as the protein concentration, pH and the ionic strength, or extrinsic conditions as temperature, low or even high molecular weight cosolutes, etc. Measured diameters for both 7S and 11S globulins protein coincided with the trimeric and monomer structures, respectively. The molecular weight was estimated from empirically determined size vs. protein mass relationships (Protein utility tool Nanosizer software, Malvern Instruments, Malvern, UK).

Thus, when FA concentration was 4.5 mM (0.2% *w*/*w*), the molecular weight increased from 504 to 626 kDa, whereas the self-association increased 3 times for 7S globulin when FA was at 7.0 mM (0.3% *w*/*w*).

For the case of 11S, the aggregation process proceeded in a more extensive way. Thus, when FA concentration was 4.5 mM (0.2% *w*/*w*), aggregates constituted by twelve units were obtained. At the highest FA concentration 7.0 mM (0.3% *w*/*w*) forty globulin units constituted the aggregates.

### 3.6. Determination of FA Concentration by Microbiology Model

#### 3.6.1. Calibration Curve for FA

**Figure 9:**
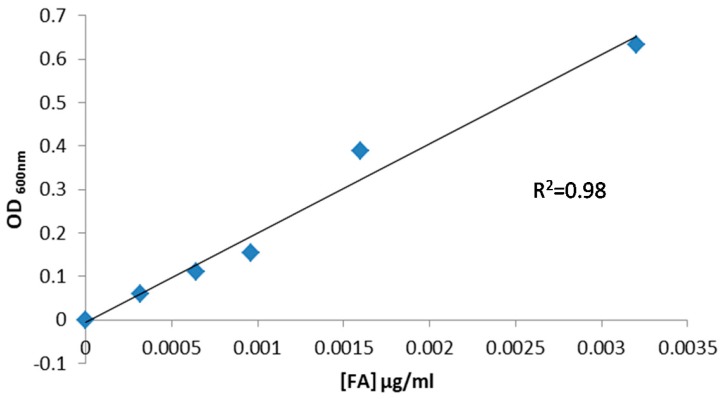
Calibration curve for *L casei* BL23 microbial growth expressed as OD at 600 nm versus FA concentration.

To determine the FA concentration, a microbiological assay was carried out using as model microorganism *Lactobacillus casei* BL23, according to Arzeni et al. [31]. This strain is auxotrophic for FA; which that must be added normally in culture media for growth. In order to determine the range of folate concentrations for which the method with *L. casei* BL23 is useful, a calibration curve was performed using free FA (Figure 9). For a concentration range between 1 × 10^−4^ and 4 × 10^−3^ μg/mL, a linear adjustment was obtained in relation to bacterial growth (measured by OD_600_) with an *R*^2^ = 0.98.

#### 3.6.2. 7S and 11S Loading Capacity

The LCFA expressed in milligrams per gram of protein represent 53 ± 9 mg FA/g protein for 11S-FA nano-complexes. On the other hand, 10 ± 0.5 mg FA/g protein was the amount found for 7S-FA nano-complexes. These values prove to be above the recommended levels of fortification [45] thus allowing the use of these nano-complexes FA in small amounts for nutraceutical preparations.

#### 3.6.3. Biological Activity of FA in Nano-Complexes

The biological activity of FA vehiculized in nano-complexes was measured in terms of the vitamin bioavailability for biomass production in *L. casei* BL23. With this purpose, nano-complexes carrying different nominal FA concentrations were added to the culture, i.e., fermentation medium. The OD_600_ values were obtained after 48 h as a measure of microbial growth. These values were compared with those systems containing free FA at the same nominal concentration. As the concentration of FA added in each culture had a nominal known concentration, the differences in microbial growth should have been caused by a different biological activity. Such differences in bacterial growth, i.e., biological activity, would be derived from globulins-vitamin complexation process. For this reason, the results of the microbiological assay were expressed as apparent concentrations of FA ([FA]_app_) and these values represent the differences in microbial growth due to a higher or lower biological activity of FA in each culture. *L casei* BL23 did not growth in culture media without FA, which was used as experimental control.

On the other hand, another set of controls were simultaneously run. Single 7S and 11S globulins were also added at the same concentrations as in nano-complexes. These controls did not show significant differences with respect to the FA free control. Table 3 shows the results for the [FA]_app_ concentrations obtained after nano-complexes addition to the culture medium. Addition of low concentrations of 11S-FA and 7S-FA nano-complexes, which contained FA concentrations equal to 3.2 × 10^−4^ μg/mL resulted in a [FA]_app_ 10 and 6 times higher than the nominal, respectively. Meanwhile, at higher nano-complexes concentrations (=1.6 × 10^−3^ μg/mL), the growth values were equivalent to the generated by the systems containing twice the FA concentration. The result presented here represents a synergistic effect concerning to the microbial growth, as *L. casei* BL23 biomass increased when FA was added to the culture media in the form of 11S and 7S globulins nano-complexes.

## 4. Discussion

FA is sensitive to some environmental factors associated with processing and storage conditions light, oxygen and heating. A way to overcome this question is to generate nano-complexes with proteins; which can act as carriers for encapsulation and protection of FA. It is well known that proteins generally exhibit a high affinity to some ligands. In this context, many models based on fluorescence were used to study the interaction between proteins and drugs [46,47]. To analyze the 7S and 11S interactions by fluorescence quenching, the complex was hardly supposed to emit it [39] even the photophysical character of the fluorophore is generally sensitive to the polarity of the surrounding environment [48]. Therefore, the total fluorescence intensity at which contributed Trp and Tyr residues, was proportional to the residue number and the hydrophobicity of their surrounding environment [49].

The reaction system containing 7S or 11S emitted fluorescence even at the maximum FA concentration added, which demonstrated that the nano-complex fluorescence was never equal zero but weaker than the single proteins. By means of analysis of fluorescence parameters, knowledge about changes in 7S and 11S globulins induced by the interaction with FA could be obtained.

Here, *the Stern-Volmer* model was applied firstly and then *the modified Double log model* in order to obtain parameters that describes globulins and FA interactions. The binding constants and the number of binding sites obtained were similar when considering these two models (Table 1). Just to clarify, Berezhkovskiy [50] raises an interesting question: the calculated number of binding sites increased with the ligand concentration as experimentally measured of unbound drug fraction. Thus, *n* could be different from the number of molecules actually bound to the sites. In macromolecules as proteins the number of molecules bound to the binding sites follows a binomial type distribution, if the number of binding sites is fixed [46]. Considering the binding process on a receptor with *n* sites of the same reaction, with the binding dissociation constant *K_d_* = 1/*K_a_*, a FA concentration ~10 × *K_d_* would be required to occupy 90% of the binding sites. Values for *K_d_* ≥ 10^3^ M^−1^ indicate that low affinity sites were scarcely occupied, in comparison with the binding sites with high affinity and they would not be detected at low FA concentrations. Then, if globulins had a low number of affinity sites the binding to them would be comparable to the binding to a single high affinity site. That would be the reason why the increase of the quantity of sites would lead to the increase of ligand bound to them. The results obtained here indicated that *n* values were practically the same which implies the presence of at least one main type of binding site for FA on 7S and 11S globulins.

The extent of ligand-induced protein fluorescence quenching has correlation with the affinity for the ligand [51]. At this respect, the order of magnitude for the values adopted by *K_a_* (10^4^–10^5^) would be indicative of strong binding interactions between FA and globulins. In comparison with other proteins used as carriers, the association constant obtained for 7S-FA and 11S-FA nano-complexes resulted similar to that for BSA-FA-binding [52] for example. This affirmation can be extended to other proteins reported in scientific literature which are used in pharmaceutical and in the food fields. For instance it was found that the FA-BSA, β-lactoglobulin (β-lg)-FA and α-lactalbumin (α-lac)-FA complexes presented similar affinity, which was about 1 × 10^5^ M^−1^ [22,49,52]; On the other hand studies made on β-casein, a polypeptide amphiphilic in nature with a strong tendency to form micelles manifested a *K_a_* of 7.0 ± 0.9 × 10^4^ M^−1^ [53]. In general terms it was considered that protein-FA interaction was mainly hydrophobically driven, specifically FA interacts with the hydrophobic pockets on the these macromolecules [22,54]. It was also reported that FA could interact via multi noncovalent interaction (including Van der Waals, hydrogen bounds and even hydrophobic forces) with proteases as evaluated by a thermodynamic approach [55]. Computational studies made by Shi et al. (2017) [36] also concluded that these forces also rule the interactions between FA and α-amylase, pepsin and trypsin. These interactions would have impact on the distribution of particle sizes, showing greater evidence of aggregation for the 11S-FA nano-complexes, as reflected by the greater increase of Z_av_ with more extensive interactions than in the case of the 7S-FA.

These results concerning to protein aggregation induced by FA are in line with those obtained by [23], who characterized the interactions between β-lg and FA. It was observed that the addition of FA affected the particle size distribution curves and doubled the average size values for samples containing nano-complexes of β-lg and FA.

When FA was encapsulated into zein nanoparticles, a moderate increase in the mean size of the resulting carriers was observed [56]. On the other hand, Arzeni et al. [31], working with egg white proteins, found that the particle size distribution of nanoparticles previously obtained by ultrasonication remained unaltered after the FA binding. However, a little increase in particle size was observed for thermosonicated nanoparticles when these were bound to FA.

The physical stability of the proteins in solution depends strongly of the attractive and repulsive interactions between the protein molecules and their interactions with co-solutes, in addition to the conformation of their secondary and tertiary structure [57]. The ζ potential is an important and useful indicator to predict and control the stability of colloidal suspensions. Both type of nano-complexes 7S-FA and the 11S-FA system showed colloidal stability as they had a net charge to values ≥|10 mV|. Scientific literature is not abundant in data referring to surface charge of proteins loaded with FA. It was informed that the original negative ζ potential decreased after FA loading on zein based nanoparticles for the oral vitamin delivery [58]. It can be concluded that the response of any protein systems is strongly dependent on its intrinsic structure and the charge modification induced by FA. The union between these globulins and FA probably only induced subtle conformational changes in the protein molecule with subtle modifications in electrophoretic mobility and consequently in the ζ potential derived values [23].

Results concerning to PDI were attributed to the aggregation phenomenon proceeded giving a limited variety of aggregates. The presence of FA led to an increase in PDI values only for 11S-FA nano-complexes at the maximum vitamin concentration used, i.e., the nano-complex particle size distribution became heterogeneous.

On the other hand, a low PDI value could be highly beneficial from a technological point of view as the nano-complexes generated by FA addition would transporter the vitamin efficiently in terms of the same amount of FA loaded.

Information about protein self-assembling phenomena induced by FA with biotechnological impact is really scarce. Nano-complex self-assembly and particle formation processes were previously observed in other protein-ligand pairs under various formulation conditions. Thus, Al-Shabib et al. [59] found that tartrazine, a synthetic azo-dye commonly used as a coloring agent in various foodstuffs, accelerated the aggregation in myoglobin under acidic pH, which proceeded via electrostatic interactions. Stirpe et al. [60] concluded that the bioactive resveratrol binds non-covalently to human serum albumin (HSA) in native state, in a specific binding site. The interactions between the bioactive and the protein occur via protein aromatic residues. Concerning green tea polyphenols, von Staszewski et al. [61] reported that these compounds induced whey protein aggregation, which was more extensive nearer to the proteins pI. The aggregates were big enough to provoke precipitation.

In microbiology, bioavailability was represented by a chemical crossing a cell membrane, entering a cell and becoming available at a site of biological activity [62]. The microbial growth could obey to a higher bioavailability of the vitamin for *L. casei* BL23 when FA was vehiculized in nano-complexes, showing that the apparent concentration exceeds the nominal value added in FACM medium. In practical terms, one could say that the bacteria “see” more FA than the single vitamin added to the culture media.

The folate transport systems of *L. casei* BL23 were well characterized by Henderson et al. and Eudes et al. [63,64]. These systems involve two small membrane proteins for specific binding substrate, one for folate and another for thiamine (FolT and ThiT respectively) and components shared by both systems. However, it is not established how FA would enter to the cell bound to proteins. To understand this phenomenon an analogy with milk proteins could be established. It is well known that *L. casei* is an auxotrophic specie for many amino acids; however, they have the ability to grow in poor media, that to say in low concentrations of free amino acid, such as milk. How is this possible? Milk contains caseins as major proteins and lactobacilli use the proteolytic system to degrade and incorporate them. The proteolytic system consists of three components depending on the function. It has been well established that casein degradation is trigger firstly by a single extracellular serine-proteinase anchored to the cell wall (PrtP), which degrades caseins into peptides. Then, the degradation products of the caseins must go across the membrane entering to the cytoplasm. For these functions, *Lactobacillus* genus has a transport system that translocates the breakdown products across the cytoplasmic membrane. Finally, the small peptides are hydrolyzed by multiple intracellular peptidases [65]. The transport system is composed of three elements the first one for di peptide (DtpT), the second one for tripeptides (DtpP) and the third one for oligopeptides (Opp). The last one can incorporate peptides of up to 18 residues. To explain the bioavailability increase of folate when it was loaded in 7S and 11S globulins, *L. casei* BL23 should incorporate the vitamin by the canonical route, i.e., via specific membrane receptors. In addition, FA would be incorporated through the proteolytic system. PrtP would trigger the degradation of 7S-FA and 11S-FA nano-complexes and then the peptides produced, would be transported jointly with the bound FA into the cell interior. This hypothesis can be supported by Piuri et al. [66], who explained the crucial function of the proteolytic regulation system. In this system, the genes involved in proteolytic activity are depressed at low concentrations of peptides. Meanwhile at high protein concentrations, the repression process starts. In line with the results exposed here, at low concentrations of nano-complexes, which means low peptide concentrations, an [FA]_app_ ten times higher than the nominal value was observed. On the other hand, at high concentrations of nano-complexes, the determined [FA]_app_ resulted only twice higher than nominal one.

## 5. Conclusions

7S and 11S globulins could be efficiently isolated from defeated soy flour. Both globulins could be complexated with FA in solution generating nano-complexes. Even though the response obtained from each globulin could be different. 11S globulin seemed to be more prone to self-assembly, i.e., more extensive aggregates formation induced by FA, as the results obtained from DLS. ζ-potential did not change significantly in comparison with the single protein.

The results derived from the *Stern-Volmer* and the *modified Double logarithm* models’ application to experimental fluorescence data manifested similar values for the constant binding and for the ligand sites parameters. The binding of FA provoked protein aggregation via self-association. The estate of association strongly depended on the FA concentration. 7S-FA and 11S-FA nano-complexes construction could be achieved. The results obtained here showed that these globulins could be effective carriers for this bioactive cargo. In turn, these nano-complexes could find applications in the technological fields of pharmaceutical and nutraceutical products. The biological activity measured as the *Lactobacillus casei* BL23 growth indicated a higher increase in biomass formation when FA was vehiculized in nano-complexes. This finding could have direct application in fermentation processes where this bacterium could take part.

## Figures and Tables

**Table 1:** Parameters derived from the Sterm-Volmer and the modified Double logarithm model application for 7S-FA and 11S-FA complexes.

	Parameters derived from stern-volmer Model			Parameters derived from modified double logarithm model		
	*n*	*K_s_* [M^−1^]	*R^2^*	*n*	*K_a_* [M^−1^]	*R^2^*
7S-FA	0.95	2.4 × 10^4^	0.9982	0.8397	4.00 × 10^4^	0.9909
11S-FA	1.13	1.87 × 10^5^	0.9693	0.9332	4.36 × 10^4^	0.9946

**Table 2:** Average diameter values (Z_ave_), ζ potential and PDI index for each size distribution of the studied systems. In Trizma buffer, pH 7, *I* = 0.05 M, 20 °C.

System (mM) *	Z_ave_ (d.nm) **	ζ potential (mV) **	PDI **
7S (5.5)	19.57 ± 1.2	−8 ± 2.0	0.404 ± 0.06
7S-FA (5.5–4.5)	22.31 ± 1.2	−12 ± 2.0	0.375 ± 0.09
7S-FA (5.5–7.0)	33.86 ± 1.4	−10 ± 2.0	0.498 ± 0.36
11S (3.0)	21.52 ± 1.5	−11 ± 2.0	0.535 ± 0.80
11S-FA (3.0–4.5)	38.16 ± 1.6	−12 ± 2.0	0.581 ± 0.09
11S-FA (3.0–7.0)	51.33 ± 1.3	−14 ± 2.0	0.851 ± 0.40

* Globulin or globulin-FA solution concentration. ** mean ± SD.

**Table 3:** First column indicates the nominal FA concentration in nano-complexes added to the culture media. The results of the [FA]_app_ obtained for 7S and 11S globulins-FA are shown. Globulin concentration equal to 2 μM and FA 5 μM.

[FA] µg/mL	11S [FA]_app_	7S [FA]_app_
3.2 × 10^−4^	3.04 × 10^−3^	1.99 × 10^−3^
6.4 × 10^−4^	2.24 × 10^−3^	2.01 × 10^−3^
9.6 × 10^−4^	2.87 × 10^−3^	2.47 × 10^−3^
1.6 × 10^−3^	2.25 × 10^−3^	2.83 × 10^−3^
